# Factors associated with amputation among patients with diabetic foot ulcers in a Saudi population

**DOI:** 10.1186/s13104-018-3372-z

**Published:** 2018-04-27

**Authors:** Imad R. Musa, Mohanned O. N. Ahmed, Elsanousi Ibrahim Sabir, Ibrahim F. Alsheneber, Elsayed M. E. Ibrahim, Gussay Badawi Mohamed, Rasha Elamin Awadallah, Tarig Abbas, Gasim Ibrahim Gasim

**Affiliations:** 1 King Abdulaziz Armed Forces Hospital, Al Dhahran, Saudi Arabia; 2 Prince Abdulaziz bin Mossaed Hospital, Arar, Saudi Arabia; 3 Faculty of Medicine, Alneelain University, Khartoum, Sudan

**Keywords:** Saudi Arabia, Diabetic foot ulcers, Complications, Amputation

## Abstract

**Objectives:**

A prospective study was conducted at the Armed Forces Hospital, Dhahran, Saudi Arabia, between January 2015 and December 2016 to identify the risk factors associated with amputation among diabetic foot ulcers DFUs patients.

**Results:**

In total, 82 patients were recruited. Fifty-five of the patients were males (67.07%), the mean (SD) age of the participants was 60 (± 11.4) years, the mean duration of diabetes was 8.5 (± 3.7) years, and the mean haemoglobin A1c was 4.8 (± 2.8)%. In Univariate analysis, older age and high white blood cell count (WBC) were factors associated with amputation (OR = 1.1, 95% CI = 1–1.1, P = 0.012; and OR = 383, 95% CI = 7.9–18,665, P = 0.003, respectively). On the other hand, an ischaemic ulcer was half as likely as a neuropathic ulcer to lead to amputation (OR = 0.5, 95% CI = 0.3–0.9, P = 0.036), and a higher Wagner’s grade was found to be protective against amputation OR = 14.5, 95% CI = 4.3–49.4, P < 0.001. In conclusion, the current study showed that although a number of factors have been described to complicate diabetic ulcers by different researchers, none of those factors were identified in our study apart from older age and high WBC.

## Introduction

Diabetes mellitus is a common endocrinopathy known for its various complications, including diabetic foot ulcers (DFUs) which often result in amputated limbs [1]. The prevalence of foot ulcers among patients with diabetes mellitus ranges from 4 to 10%, and its lifetime incidence may reach up to 25% [2]. Conservative management of DFU may be affected by proper offloading of the wounds, correct daily foot hygiene, and impaired distal vascular flow. Treatment of a DFU is difficult; it frequently gets infected, and it is therefore a very common cause of hospitalization [3]. Diabetes mellitus increases the risk of lower extremity amputations (LEAs) by up to 56% over 5 years, and the bulk of these amputations are reported to be predated (up to 85%) by a poorly healing ulcer [4]. It is predicted that diabetes-related LEAs will remain a significant source of morbidity and mortality, taking into consideration the rapid global growth of the diabetic population and the high incidence of DFUs [5]. The Global Lower Extremity Study Group defines LEA as the complete loss of any part of the lower limb regardless of the cause [6]. Nearly 82% of LEAs occur among diabetics suffering foot ulceration [7]. The track to ulceration and finally LEA may include essential contributions from background diabetes-related pathophysiology (neuropathy, peripheral arterial disease (PAD), foot deformity and impaired joint mobility), initiating environments (trauma), infection, and complicated healing [8]. The indications for LEA are varied and include severe soft-tissue infection, osteomyelitis, the presence of peripheral arterial occlusion, and gangrene. The impact of LEA surgery on an individual patient is substantial, and therefore amputation is always the last resort for any unsalvageable limb [9]. Regardless of its cause, the avoidance of amputation should be attempted once a DFU has developed or been identified in the hospital [4, 5]. DFUs follow a common progression, starting with a small ulcer or a surgical wound. The bulk of DFUs (60–80%) heal, while 10–15% of them continue to be active, and up to 24% of DFUs eventually lead to LEAs [4, 8]. Previous research has shown that the duration of diabetes mellitus [10], a history of previous amputations or foot ulceration [11], inadequate glycaemic control [12], a history of hypertension [13], hyperlipidaemia [13], the presence of PAD [13], a history of peripheral neuropathy [14], a history of osteomyelitis [15], and wound severity [16] are independent risk factors for LEA. Furthermore, old age [17], a history of smoking [18], anaemia [19], an elevated white blood cell count [20], hypoalbuminemia [19], and the presence of microvascular [15, 21] and macrovascular complications [22, 23] are all believed to contribute to these risk factors. Nevertheless, different studies have reported different findings, and the published data identifying such predictors for diabetes-related LEA in Saudi Arabia are scarce. Considering the previous facts about DFUs along with their great burden in Saudi Arabia, where its prevalence ranges from 11.4 to 29.7% [24, 25]. The current study was conducted to identify the factors associated with amputation among patients with DFU in a Saudi population.

## Main text

### Methods

A prospective study was conducted to collect data from patients presenting with diabetic foot ulcers to King Abdul Aziz Armed Forces Hospital in Dhahran City, Saudi Arabia, between January 2015 and December 2016, where they were treated and followed based on a standardized protocol until the wound healed or the patient died. Patients who underwent revascularization were excluded. All lesions were evaluated by the same team to minimize assessment discrepancies. After obtaining the written informed consent of the patients, the data were collected using a standardized, pretested questionnaire that included demographic data (age, gender, marital status, residence, and education level), history of smoking, history of alcohol consumption, duration of diabetes, The outcome of the DFU(whether the patient was amputated or not) haematological and biochemical profile at the first admission (complete blood count, blood glucose, haemoglobin A1c level, lipid profile, kidney profile, and Wagner’s grade).

For operational purposes, a diabetic foot ulcer was defined as a breach of the normal skin manifesting as an induration, ulceration or change in foot colour lasting for 2 weeks or more in a known diabetes mellitus patient (DM) or an individual recently diagnosed with DM. Amputation was documented if it resulted from surgery, spontaneously or both in a patient with DFU.

### Ethical approval

Ethical approval for the study was obtained from the ethical committee at the Armed Forces Hospital at King Abdulaziz Air Base in Al Dhahran.

### Statistical analysis

After data entry, the SPSS (version 20) statistical package was used for data analysis. After checking for the normality of the distribution of the data, non-normally distributed data were log-transformed, and then the means and standard deviations were calculated. Univariate analysis was performed to identify factors associated with amputation in patients with DFUs. The P value was considered significant if it was less than 0.05.

### Results

In total, 82 patients were recruited. Fifty-five of the patients were male (67.07%), and the mean (SD) age of the participants was 60 (11.4) years. The mean duration of diabetes was 8.5 (3.7) years. These patients showed a mean haemoglobin A1c of 4.8 (2.8)%. Thirty-three patients (40.24%) underwent amputation by the end of the study. Among this group of patients who underwent amputation, 2 were Wagner’s grade 1, 14 were Wagner’s grade 2, 11 were Wagner’s 3, and the remaining 6 were grade 4 when they started the study; none of the ulcers were Wagner’s grade 5 (Table 1). Univariate analysis showed that older age and high WBC were factors associated with amputation (OR = 1.1, 95% CI = 1–1.1, P = 0.012; and OR = 383, 95% CI = 7.9–18,665, P = 0.003, respectively). On the other hand, an ischaemic ulcer was half as likely as a neuropathic ulcer to lead to amputation (OR = 0.5, 95% CI = 0.3–0.9, P = 0.036), and a higher Wagner’s grade was found to be protective against amputation OR = 14.5, 95% CI = 4.3–49.4, P < 0.001 (Table 2).

**Table 1:** The characteristics of the study population

Variable	Not amputated	Amputated
Gender (number of patients)		
Male	31	24
Female	18	9
Age, mean (SD) (years)	60.0 (11.4)	66 (12.6)
Duration of diabetes, mean (SD) (years)	8.5 (3.6)	6.8 (4)
Haemoglobin A1c, mean (SD)	4.8 (2.8)	3.8 (2.8)
Triglycerides, mean (SD)	1.7 (1.9)	1.8 (1.7)
Cholesterol, mean (SD)	3.8 (1.3)	4.2 (1.1)
Haemoglobin, mean (SD)	12.6 (1.2)	10 (2.3)
Total white blood cell count, mean (SD) (cells/cm^2^) × 10^3^	10 (0.0015)	12.6 (0.0016)
Type of ulcer (number of patients)		
Ischaemic	21	8
Neuropathic	11	13
Mixed	17	12
Other comorbidities	18	11
History of hypertension	39	21
History of smoking	4	6
Wagner’s grade		
Grade 1	9	2
Grade 2	14	14
Grade 3	11	11
Grade 4	12	6
Grade 5	2	0

**Table 2:** Univariate analysis of the predictors of diabetic foot ulcer outcome among a Saudi population

Variable	OR	CI	P value
Gender (number of patients)			
Male	0.3	0.1–1.1	0.076
Female			
Age, mean (SD) (years)	1.1	1–1.1	0.012
Duration of diabetes, mean (SD) (years)	0.7	0.3–1.7	0.402
Haemoglobin A1c, mean (SD)	0.6	0.2–1.8	0.318
Triglycerides, mean (SD)	1.3	0.1–24.7	0.859
Cholesterol, mean (SD)	1.3	0.7–2.6	0.388
Haemoglobin, mean (SD)	0.004	0.0–1.9	0.076
Total white blood cell count, mean (SD) (cells/cm^2^) × 10^3^	383	7.9–18,665	0.003
Type of ulcer (number of patients)			
Ischaemic			
Neuropathic	0.5	0.3–0.9	0.036
Mixed			
Other comorbidities	0.3	0.1–0.9	0.026
History of hypertension	0.5	0.1–1.7	0.249
History of smoking	1.2	0.2–6.2	0.833
Wagner’s grade			
Grade 1			
Grade 2			
Grade 3	14.5	4.3–49.4	< 0.001
Grade 4			
Grade 5			

### Discussion

Published data about diabetic ulcers in Saudi Arabia is scarce, and the current report is one of the few studies discussing this important issue. The mean age of the study participants was 60 (± 11.4) years, which is a younger age than in the study by Altawfiq et al., where the mean age was 64.8 years [26]. The probable explanation for this is the worldwide metabolic syndrome epidemic, which is particularly affecting the Arabian Gulf area. This epidemic is tending to hit more people at a younger age. Al-Rubeaan reported in their large cohort study that age ≥ 45 years is a risk factor for developing diabetic foot ulcers in a Saudi population [27]. Univariate analysis has shown a number of factors that predict amputation, namely older age and higher WBC counts. These findings have been reported in national Saudi studies as well as in international studies with large cohorts [27, 28]. An older age is likely to be associated with a poorer outcome probably because of a slower immune response to infection and the presence of other comorbidities that delay healing such as an impaired vascular blood flow. On the other hand, a longer duration of diabetes is likely to be associated with more diabetic complications such as micro and macrovascular complications, which are likely to play crucial roles in the skin breach and the perpetuation of the ulcer. Although the finding of an association between low HbA1c and amputation in the current study seems unusual, considering the mean age of the group as well as their diabetics status, it is likely that such patients suffer renal impairment, which is reflected in deceptively better glycaemic control. It is not surprising to find an association between increased total WBCs and an increased frequency of amputation, since the former is known to be associated with poorer glucose metabolism and infection. However, in our setting, it is obvious that the sample size lacks enough power to recognize increased TWBCs as a risk factor. In line with other studies, the current study found that an ischaemic ulcer is less likely to lead to an amputation than a neuropathic ulcer [27]. Likewise, the current study showed that a higher Wagner’s grade was associated with an increased incidence of amputation if compared to the lower grades, a finding that has been shown by different studies. Our findings were further supported by a group of Kenyan researchers in their cross sectional study among more than one thousand diabetics with diabetic foot ulcers [29].

### Conclusion

The current study concludes that although a number of factors have been described to complicate diabetic ulcers by different researchers, none of those factors were identified in our study apart from older age and high WBC.

## Limitations

We acknowledge that the sample size is rather small, as reflected by the wide confidence intervals. Moreover, despite the importance of vascular assessment, we were not able to include it in our analysis.

## Abbreviations

DFU: diabetic foot ulcer
WBC: white blood count
HbA1c: haemoglobin A1c
LEA: lower extremity amputation
PAD: peripheral arterial disease
